# Arsenic Exposure and Type 2 Diabetes: MicroRNAs as Mechanistic Links?

**DOI:** 10.1007/s11892-017-0845-8

**Published:** 2017-03-08

**Authors:** Rowan Beck, Miroslav Styblo, Praveen Sethupathy

**Affiliations:** 1 Department of Genetics, School of Medicine, University of North Carolina at Chapel Hill, Chapel Hill, NC 27599 USA; 2 Curriculum in Genetics and Molecular Biology, University of North Carolina at Chapel Hill, Chapel Hill, NC 27599 USA; 3 Department of Nutrition, Gillings School of Global Public Health, University of North Carolina at Chapel Hill, Chapel Hill, NC 27599 USA

**Keywords:** Diabetes, Arsenic, MicroRNAs, β-Cell, Insulin, Metabolism

## Abstract

**Purpose of Review:**

The goal of this review is to delineate the following: (1) the primary means of inorganic arsenic (iAs) exposure for human populations, (2) the adverse public health outcomes associated with chronic iAs exposure, (3) the pathophysiological connection between arsenic and type 2 diabetes (T2D), and (4) the incipient evidence for microRNAs as candidate mechanistic links between iAs exposure and T2D.

**Recent Findings:**

Exposure to iAs in animal models has been associated with the dysfunction of several different cell types and tissues, including liver and pancreatic islets. Many microRNAs that have been identified as responsive to iAs exposure under in vitro and/or in vivo conditions have also been shown in independent studies to regulate processes that underlie T2D etiology, such as glucose-stimulated insulin secretion from pancreatic beta cells.

**Summary:**

Defects in insulin secretion could be, in part, associated with aberrant microRNA expression and activity. Additional in vivo studies need to be performed with standardized concentrations and durations of arsenic exposure in order to evaluate rigorously microRNAs as molecular drivers of iAs-associated diabetes.

## Introduction

Type 2 diabetes (T2D) is a complex metabolic disorder characterized by hyperglycemia that is generally caused by defects in insulin production, secretion, and/or systemic action. While both genetics and lifestyle components, such as diet and exercise, can significantly increase risk for T2D, chronic exposure to chemical diabetogens is less studied in the context of T2D etiology. Inorganic arsenic (iAs) is one such environmental diabetogen. The Environmental Protection Agency (EPA) and the Agency for Toxic Substances and Disease Registry (ATSDR) rank arsenic as first on the US Priority List of Hazardous Substances. Over 300 million people across more than 70 countries are exposed to iAs in their drinking water, and chronic exposure to iAs is associated with numerous adverse health effects including cancer, cardiovascular disease, hypertension, and, notably, diabetes.

Though the exact mechanism by which arsenic influences metabolic disorders is unknown, dysregulation of microRNAs (miRNAs) has emerged as a potential mode of action. miRNAs are short, non-coding molecules that negatively regulate gene expression at the post-transcriptional level. They are involved in the control of metabolic processes associated with impaired glucose tolerance and diabetes, such as gluconeogenesis in the liver and insulin secretion from pancreatic beta cells. The identification of miRNAs as a potential mechanism for the development and progression of iAs-associated diabetes could open a new avenue for therapeutic options.

## Sources of iAs Exposure

Sources of human exposure to iAs are both natural and anthropogenic. Arsenic is a naturally occurring metalloid and is present mainly as a sulfide in over 200 mineral species containing a mixture of metals, including silver, lead, copper, nickel, antimony, cobalt, and iron [1, 2]. Arsenic is released into air, water, and soil as a result of volcanic activity, leaching of arsenic from soil to groundwater, and industrial processes [1]. Approximately one-third of the global atmospheric flux of arsenic (7900 t/year) is estimated to be from natural sources with volcanic activity being the most significant contributor [1, 2]. The rate of its release from minerals can be enhanced by mining activities, exposing the minerals to weathering processes during excavation and leading to the accumulation of iAs in soil and water. iAs is readily metabolized by microorganisms, plants, and animals into organoarsenic species, including volatile arsenicals that can enter the atmosphere [1, 2].

Arsenic and arsenic-containing compounds have been produced and used commercially for centuries [3]. Major anthropogenic sources of arsenic are associated with mining, smelting of non-ferrous metals, and burning of fossil fuels, which lead to the contamination of air, water, and soil. Furthermore, the historical use of arsenic-containing pesticides and herbicides as well as the use of arsenic in the preservation of timber has left large tracts of agricultural land contaminated [1, 2]. Past and ongoing uses of arsenic include pharmaceuticals, wood preservatives, agricultural chemicals, and applications in the mining, metallurgical, glass-making, and semiconductor industries [3]. In most regions though, drinking water is the most common source of iAs for humans [4], followed by agricultural products contaminated with iAs or organorasenicals.

Once ingested, iAs is methylated to either trivalent iAs^III^- or pentavalent iAs^V^-containing metabolites, including methylarsenite (MAs^III^), dimethylarsenite (DMAs^III^), methylarsenate (MAs^V^), and dimethylarsenate (DMAs^V^) [5–9]. A critical enzyme in this process is arsenic (+3 oxidation state) methyltransferase (AS3MT), a member of the large superfamily of *S*-adenosylmethionine (SAM)-dependent enzymes [10]. These reactions appear to occur irrespective of whether the means of exposure is inhalation, ingestion, or a parenteral route [3]. The methylated arsenic species are more readily excreted in the urine, resulting in lower tissue retention of iAs. Therefore, the methylation of iAs has been viewed historically as a detoxification process [11–13]. This notion, however, is being called into question, as there have been recent studies showing that the trivalent methylated species, MAs^III^ and DMAs^III^, are more toxic and biologically active than unmethylated iAs in laboratory models [14, 15]. In addition, growing evidence suggests that MAs^III^ and DMAs^III^ also contribute to the adverse health effects of chronic iAs exposure in humans [16••, 17–19].

## Arsenic Exposure and Public Health

The Environmental Protection Agency (EPA) and the Agency for Toxic Substances and Disease Registry (ATSDR) rank arsenic as first on the US Priority List of Hazardous Substances [20]. Both the inhalation and ingestion of iAs have been linked to an increased risk of cancer of the lungs, urinary bladder, kidney, skin, liver, and prostate [21]. Several studies have revealed an elevated cancer risk for populations exposed to varying amounts of iAs from industrial emissions [4, 21–23]. In addition, an association between various cancers and contaminated drinking water [21] has been observed in cohorts from a variety of regions around the world including Taiwan [24], Japan [25], USA [26], and parts of South America [27, 28]. Recent reports from the United Nations International Children’s Emergency Fund (UNICEF) indicate that over 140 million people across more than 70 countries are exposed to iAs in their drinking water [29]. In the USA alone, there are over 13 million people drinking water containing iAs at levels higher than the current EPA maximum contaminant level of 10 μg As/L, i.e., ten parts per billion (ppb) [20]. While most dietary arsenic is derived from saltwater fish and seafood [30, 31], only a small proportion occurs in the inorganic form [32, 33]. Significant amounts of iAs are absorbed when agricultural plants are grown in or watered with iAs-contaminated water, and so the most abundant sources of dietary iAs include rice, grains, and flour [34]. Chronic exposure to iAs has been associated with numerous adverse health effects in addition to cancer, including cardiovascular disease [35, 36], hypertension [37, 38], and recently, T2D [39].

Type 2 diabetes is a complex metabolic disorder characterized by hyperglycemia that is generally caused by defects in insulin production, secretion, and/or systemic action [19]. T2D can lead to numerous long-term complications such as cardiovascular disease, nerve and kidney damage, chronic inflammation, or diabetic ketoacidosis [19]. Risk factors for T2D include both genetic and environmental variables [40, 41]. Over 80 distinct genetic loci have now been identified across diverse human populations that significantly increase risk for T2D [42]. While lifestyle components such as diet and exercise are very important contributing factors, particularly given that obesity often precedes some forms of T2D, chronic exposures to environmental chemicals are less studied in the context of T2D etiology.

The first studies of an association between arsenic exposure and T2D took place in Europe in the mid-1990s and were focused on occupational exposure [43–45] to iAs, as well as in Taiwan and Bangladesh in the late 1980s to mid-1990s, which centered on iAs exposure through drinking water [46–48]. Findings in other studies were inconsistent [37, 49, 50], possibly due to variation in exposure measurements and lack of standardized diagnostic criteria, especially for populations with low-to-moderate iAs levels in drinking water (<150 ppb). Therefore, the association with T2D was viewed as ambiguous at best. However, several of these studies assessed iAs exposure indirectly by measuring arsenic levels in drinking water sources rather than using biomarkers of exposure [46, 48]. Also, some of these studies ascertained diabetes status based on self-reporting or death certificates [43, 51]. More recent studies that use direct, quantitative methods to measure iAs exposure and that include specific clinically relevant criteria for diabetes, consistently find a significant association with diabetes at even low-to-moderate exposure levels [52–58]. For a thorough summary of these studies, we refer the reader to the following review articles [59••, 60, 61]. Notably, a review by the National Toxicology Program concluded that while further research is needed, recent studies of low-level exposure using improved measures of exposure and outcome support an association between arsenic and diabetes [59••]. With a growing appreciation for the epidemiological connection between iAs exposure and T2D, it is of substantial interest to determine whether, and the means by which, iAs contributes to T2D pathophysiology.

## Arsenic and T2D Pathophysiology

Laboratory studies have shown that 48-h exposure to 2 μM of iAs^III^, or 0.1 μM of MAs^III^ or DMAs^III^, is sufficient to impair the in vitro function of pancreatic islets isolated from C57BL/6 mice as measured by glucose-stimulated insulin secretion (GSIS) [18]. In two other studies, 5 μM of sodium arsenite (iAs^III^) for 72 h in primary rat pancreatic β-cells reduced insulin mRNA expression [62] and 0.5 μM sodium arsenite in a rat insulinoma cell line suppressed Ca^2+^ influx, thereby inhibiting insulin vesicle packaging and impairing GSIS [63]. In a separate report, a 96-h, 0.25-μM iAs^III^ treatment of a rat insulinoma (INS-1-832/13) cell line was shown to induce a significant Nrf2-mediated antioxidant response, which suppresses endogenous reactive oxygen species that are thought to be involved in insulin secretion [64].

Aside from pancreatic β-cell defects, iAs^III^ exposure has been shown to inhibit differentiation of fat cells, or adipocytes, which play a major role in glucose utilization and energy homeostasis. Specifically, 3T3-L1 pre-adipocyte cells treated in vitro with 6 μM iAs^III^ for 2 months decreased expression of PPARγ [65], which drives adipocytic differentiation [66]. Impaired PPARγ signaling in adipose can lead to reduced insulin sensitivity [65]. iAs^III^ and its trivalent methylated metabolites [17] were also shown to inhibit insulin signaling and insulin-stimulated glucose uptake in a mature mouse adipocyte (3T3-L1) cell line in culture [67, 68].

In the liver, 3 mg/L of sodium arsenite enhances gluconeogenesis in both normo-glycemic C57BLKS/J (db/m) and diabetic C57BKS/Lepr^db^ (db/db) mice, at least in part by increasing levels of protein tyrosine phosphatase-1B (Ptp1b) [69], which is a known suppressor of hepatic insulin signaling [70]. Another study in estrogen-deficient ICR/HaJ mice reported that 0.05 ppm iAs in drinking water for 6 weeks can stimulate the transcription of phosphoenolpyruvate carboxykinase (*Pepck*) transcription and thereby likely promote hepatic glucose production [71].

Lastly, iAs is a potent endocrine disruptor of estrogen receptor (ER)-mediated gene regulation and has been shown to alter steroid hormone receptor (SR)-mediated signaling at very low, environmentally relevant concentrations in both cell culture and whole-animal models [72–74]. These receptors play a critical role in normal biology and development [75], including energy balance and glucose homeostasis [76]. Though the exact mechanisms of action remain unknown, multiple studies support an association between endocrine disruptors and metabolic syndrome [77, 78], which may partially explain how chronic exposure to iAs has been associated with pathophysiological illnesses such as T2D.

Taken together, these studies show that the epidemiological link between iAs exposure and T2D may be mediated in part by iAs-exposure-associated defects in several different cell types and tissues, including islets, adipose, and liver, leading to either impaired insulin secretion or insulin resistance. For more details on these studies, we refer the reader to the following reviews [59••, 79, 80]. The underlying molecular mechanisms of these defects remain poorly characterized and merit deeper investigation.

## Candidate Mechanisms Underlying Effects of Arsenic on Diabetes Pathways

The precise mechanisms by which arsenic affects T2D-relevant pathways remain unclear. Several studies have observed reproducible changes in gene expression upon iAs exposure in lymphocytes (human), macrophages (human), and liver (mouse) [81–83]. The latter study in mouse liver also reported systematic changes in DNA methylation profiles upon iAs exposure. Recently, an association between levels of iAs exposure and DNA methylation patterns has been reported in studies of human populations as well [84–87]. For example, a study of iAs-exposed human populations in Bangladesh reported a positive association between global hypermethylation of peripheral blood leukocyte (PBL) DNA and iAs concentrations in urine and plasma in individuals with plasma folate concentrations >9 nmol/L [87, 88]. These findings were supported by a study of a Mexican cohort, in which the promoters of 183 genes were differentially methylated in individuals exhibiting iAs-associated skin lesions; most of the affected genes have known links to cardiometabolic disorders [84].

Altered DNA methylation status can influence transcription of not only nearby protein-coding genes, but also non-coding RNAs (ncRNAs). In more recent years, one particular class of ncRNAs, microRNAs (miRNAs), has emerged both as responsive to iAs exposure and, independently, as candidate drivers of phenotypes in metabolic disorders such as T2D [86, 89–98]. Researchers in this field are just beginning to investigate whether miRNAs may serve as mechanistic links between arsenic exposure and diabetes. Given the heightened interest in miRNA-based diagnostic and therapeutic strategies [99–103], this area represents highly translational research that could yield findings of potential clinical utility.

## MicroRNAs: Potential Mechanistic Links

miRNAs are short, non-coding RNA molecules about 22 nucleotides in length that are transcribed predominantly by RNA polymerase II and negatively regulate gene expression at the post-transcriptional level [104]. At present, more than 1000 miRNAs have been identified in the human genome. Over the last decade, it has become apparent that miRNAs play a crucial role in diverse biological processes predominantly through the fine-tuning of gene networks [105–107]. miRNAs reside either in protein coding genes where they are sometimes transcribed along with the host gene or can be found in non-protein coding regions with their own independent transcription units. Transcription leads to a primary miRNA transcript (pri-miRNA), which can range anywhere from a few hundred base pairs (bp) to hundreds of kilobases in length [108]. In the canonical miRNA biogenesis pathway, stretches of sequence in the pri-miRNA that form hairpin-like secondary structures are recognized and excised in the nucleus by the microprocessor complex involving DGCR8 and DROSHA. The resulting sequence, known as a precursor miRNA (pre-miRNA), is approximately 70–100 nucleotides in length and is exported from the nucleus into the cytoplasm by Exportin 5-Ran-GTP [104]. Once in the cytoplasm, Dicer cleaves the end of the pre-miRNA to produce a double-stranded RNA duplex about ∼22 base pairs in length. One or both of the strands is independently loaded onto the RNA-induced silencing complex (RISC), at which point the miRNA is ready to guide and tether the main effector protein in RISC, Argonaute (Ago), to target RNA sequences [104]. The stability of binding of the miRNA-RISC to a target depends in part on the extent of sequence complementarity between the miRNA and the target RNA. Once Ago is tethered to a target mRNA, it confers gene silencing by translational repression and/or mRNA degradation.

miRNAs can also be secreted into circulation and serve as plasma biomarkers [109, 110] of disease [111, 112]. The stability of miRNAs in circulation arises from the fact that they are protected by microparticles, including exosomes and lipoproteins. Changes in the levels of circulating miRNAs can be predictive of disease onset and/or disease subtype/severity, which could lead to early diagnosis and improved treatment.

In recent years, researchers have studied changes in tissue miRNA expression in response to external stimuli, including environmental toxicants [107, 113, 114•]. Arsenic and other compounds have been associated with altered miRNA expression both ex vivo and in vitro [97, 107, 115]. For example, in the peripheral blood of steel workers, the levels of two miRNAs linked to tumor progression, miR-222 and miR-21, were associated with the level of exposure to a combination of arsenic, iron, lead, and other metals in particulate matter [116]. A recent literature review by Sollome et al. indicated that the levels of over 20 additional miRNAs have been reported as being altered by varying amounts of arsenic exposure in several different cell models [107]. These results suggest that arsenic and other co-occurring environmental toxins can work alone or cooperatively to modulate the expression of miRNAs.

miRNAs in a number of metabolic tissues, including liver, adipose, and islets, have been linked previously to the pathogenesis of diabetes [117•]. Several miRNAs have been implicated in the regulation of insulin signaling in the liver, adipose, and skeletal muscle, as well as insulin production and secretion in the pancreatic islets [118–124]. Five prominent examples from the literature are described below:

- In vivo studies in mice have revealed that miR-29 controls both lipogenic and insulin signaling pathways in the liver [100, 125]. miR-29 has also been shown to respond to glucose and regulate insulin secretion in rodent β-cell-like lines, potentially in part through regulation of monocarboxylate transporter 1 (*Mct1*) [126].
- Knockout and over-expression studies in mice showed that miR-7 regulates a molecular network that controls insulin granule exocytosis and pancreatic β-cell identity [127]. Moreover, miR-7 appears to play a role in β-cell adaptation during the development of diabetes.
- miR-24 was identified as a master regulator of β-cell proliferation and insulin secretion in vitro, in large part via its direct control of Hnf1a and Neurod1 [128], encoded by two different genes in which specific mutations are known to cause maturity-onset diabetes of the young (MODY) [128].
- miR-375 was first shown to suppress GSIS in the MIN6 mouse β-cell-like cell line in part by targeting and repressing myotrophin (*Mtpn*) [119]. Subsequent studies revealed that miR-375 knockout mice exhibit altered pancreatic α-cell to β-cell ratios, increased fasting and fed plasma glucagon levels, and increased gluconeogenesis and hepatic glucose output [120].
- In separate studies, miR-34a was shown in obese mice to drive metabolic dysfunction in both liver and adipose by suppressing the gene sirtuin 1 (*Sirt1*) and also by inhibiting fibroblast growth factor 19 and 21 (Fgf19 and Fgf21) signaling [101, 102, 129]. These reports suggested that miR-34a may be an attractive therapeutic target for obesity and related metabolic diseases.

Notably, most of these miRNAs have been identified as being responsive to arsenic in different tissues and cell lines (Table 1). For example, both miR-24 and miR-29 were shown to be significantly upregulated in human umbilical vein endothelial cells (HUVEC) after treatment with 20 μM iAs for 24 h [107]. miR-29a was also altered in HepG2 hepatoma cells after treatment with 2 μM arsenic trioxide 24 h [95]. Also, in another study, 0.5 μM arsenite treatment of keratinocytes for 24 h led to an aberrant elevation of miR-34a [132].

**Table 1:** Reports in the literature of altered miRNA expression in various cell lines and tissues upon treatment or exposure to arsenic

Target	Regulation	Arsenic species	Concentration	Duration	Cells/tissue	Author
In vitro studies						
miR-190	Up	Arsenic chloride	10, 15, and 20 μM	6–12 h	Human bronchial epithelial cells	Beezhold et al. 2011 [130]
miR-21, let-7, 130a, 103, 107, 132, 16, 182, 193a-3p, 194, 196b, 19b, 200a, 215, 221, 23b, 26a, 29b, 29c, 335, 365, 493, 151-5p, 138, 301a, 96, 429, 10a, 524-3p, 487b, 361-5p, 15b, 24, 30b, 425, 532-5p, let-7a, 20b, 100, 106b, 148b, 17, 30d, 15a, 93, 125b, 101, 92a, 1184, 10b, 145, 128, 362-5p, 937, 140-3p, 20a, 25, 33a, 940, 886-3p, 874, 28-5p, 181a, 339-3p, 29a, 30c, let-7f, let-7d, let-7g, 1287, 27a, 375, 31, 191	Up	Arsenite	20 μM	24 h	HUVEC cells	Li et al. 2012 [131]
miR-299-3p, 325, 200c, 622, 585, 934, 183, 761, 892b, 122, 135a, 640, 549, 508-5p, 638, 1249, 1299, 205, 1252, 548n, 198, 617, 890, 542-5p	Down					
miR-30d, 142-5p, 150, 181a, 221, 222, 638, 663	Up	Sodium arsenite	2 μM	24 and 144 h	Human Jurkat T cell line	Sturchio et al. 2014 [94]
miR-150, 181a, 142-5p	Down			144 h		
miR-34a	Up	Arsenite	0.5 μM	24 h	Keratinocytes	Herbert et al., 2014 [132]
miR-24, 29a, 30a, 210, 886-3p	Up	Arsenic trioxide	2 μM	24 h	Hep-G-2 cells	Meng et al., 2011 [95]
miR-744, 296-5p, 663, 675	Down					
miR-215, 125b, 335, 193b, 126, 1, 125a-5p, 19a, 16, 122, 218, 100, 146a, 10b, 143, 150, let-7g, 193a-5p, 96, 183, 181b, 146b-5p, 9, 21, 27a, 98, let-7d, 148a, 155, 7, 30c, 363, 181d, 196a, 18a, 184, 29a, 200c, 10a, 144, 203, let-7a, 140-5p, 132, 23b, 148b, 124, 32, 128a, 20b, 214, let-7f, 133b, 17, 199a-3p, 181c, 378, 181a, 27b, 191	Up	Arsenic trioxide	2 μM	48 h	NB4 cells	Ghaffari et al., 2012 [96]
miR-34c-5p, 149, 212, 372	Down					
miR-200a, 200b, 200c	Down	Sodium arsenite	1 μM	8–10 months	HUC1 cells	Michailidi et al., 2015 [133]
miR-22, 21, 34a, 205, 141, 1260, 720, 1280, 200a, 19b, 29b, 27a, 1274a, 181a, 1469, 19a, 183, 101	Up	Sodium arsenite	0.5 μM	4 weeks	Human keratinocyte HaCaT cell line	Gonzalez et al., 2015 [134]
miR-1285, 34b	Down					
miR-222*	Up	Arsenic trioxide	4 μM	24 h	T24 cell line	Cao et al., 2010 [135]
miR-19a	Down					
In vivo studies						
(rno) miR-183, 872, 148b, 151, 126a, 192, 25, 532, 331, 194-1, 497, 99b, 96, 450a, 672	Up	Sodium arsenite	0.1, 1, 10, and 100 mg/L	60 days	Liver from Sprague Dawley rats	Ren et al., 2015 [136]
(rno) miR-26a, 34c, 423, 702, 6321, 20a, 425, 664-1, 125b-1, 19a, 339	Down					
Human population studies						
miR-21, 221	Down	Arsenic	Average: 54.2 (cohort 1) and 59.4 (cohort 2) nM/mM creatinine		Urine	Kong et al. 2012 [137]
Let-7a, miR-16, 20a, 20b, 26b, 96, 98, 107, 195, 454	Up	Inorganic Arsenic	Average: 64.5 μg/L (as measured in urine)		Cord blood	Rager et al. 2014 [138]

1 μM = 75 ppb [60]

While miRNAs are the drivers of several key metabolic processes associated with diabetes, such as insulin secretion and gluconeogenesis, no research has yet conclusively interconnected arsenic, miRNAs, and T2D. However, the overlap between the miRNAs modulated in expression by arsenic exposure and those involved with β-cell dysfunction and/or insulin signaling suggest that the effects of arsenic and its metabolites on the development of diabetes could be mechanistically explained, at least in part, by miRNAs and therefore warrants further investigation.

## Conclusions and Next Steps

Data from recent studies support an association between iAs exposure and diabetes. While the underlying molecular mechanisms remain poorly characterized, miRNAs have emerged as one compelling class of molecules that may serve as a mechanistic link. Future studies must address several limitations and knowledge gaps, two of which we describe here.

1. Functional studies of arsenic effects. Published in vitro and in vivo studies on arsenic exposure vary widely in terms of the arsenic species used, the concentration applied, the duration of treatment, and the cell types/tissues interrogated for functional assessment. In order to gain a more coherent understanding of the adverse diabetogenic effects of iAs, it will be important to identify and standardize a range of physiologically relevant concentrations and exposure durations. Furthermore, it will be critical to expand the cell types analyzed to include those of particular relevance to T2D etiology (e.g., intact islets, hepatocytes, intestinal epithelial cells, skeletal muscle cells). Finally, more in vivo studies, ideally across different strains of mice, are required in order to understand the effects of chronic iAs exposure in genetically diverse populations.
2. MiRNA profiling in response to iAs. Most miRNA expression studies in response to iAs exposure/treatment have been carried out with real-time quantitative PCR (RT-qPCR) and/or microarray technology. The former is low-throughput and the latter suffers from several limitations, particularly in terms of distinguishing among functionally distinct miRNAs with very similar sequences. Although not without its own challenges, sequencing-based approaches have become the gold standard for miRNA profiling [139]. It will be imperative in the future to apply sequencing technology to study the miRNA response to iAs exposure/treatment.

Future studies should focus on identifying the miRNAs that are most reproducibly altered by iAs and determining through loss- and gain-of-function studies which if any of these miRNAs may mediate the adverse effects of iAs exposure. If specific miRNAs are identified as mechanistic links between iAs and metabolic defects, then they may represent attractive therapeutic targets for iAs-associated diabetes.
